# Sin1–mTORC2 signaling drives glycolysis of developing thymocytes

**DOI:** 10.1093/jmcb/mjy078

**Published:** 2018-11-29

**Authors:** Hongbo Chi

**Affiliations:** Department of Immunology, St. Jude Children’s Research Hospital, Memphis, TN, USA

Emerging studies highlight the importance of metabolic reprogramming in T cell development and function, although how these processes are regulated remains to be fully understood. Recent advances in dissecting the roles of Sin1–mTORC2 signaling in early thymocyte development provide new insight into the dynamic interplay between immune signaling and cell metabolism, as well as the crucial role in directing thymocyte proliferation and development.

T cell metabolism is shaped by immune signals (antigens and co-stimulatory factors) and diverse environmental cues, including cytokines, growth factors, hormones, and nutrients (Zeng and Chi, 2013; Buck et al., 2015). Specific metabolic programs are tightly controlled in different stages of thymocyte development. Glycolysis is augmented by NOTCH and other signals during thymocyte development at the double-negative (DN) to double-positive (DP) transitional stage, whereas oxidative phosphorylation (OXPHOS) is active in other stages such as the quiescent DP cells (Ciofani and Zuniga-Pflucker, 2005; Yang et al., 2018). How the metabolic switch is dynamically regulated in T cell development remains poorly defined.

The mammalian target of rapamycin (mTOR) integrates environmental cues by functioning via two distinct complexes, mTOR complex 1 (mTORC1) and mTORC2 (Su and Jacinto, 2011). Both mTOR complexes play important roles in the metabolic control of T cell development. Loss of the mTORC1 signature component Raptor impairs the development of αβ T cells while promoting γδ T cells (Yang et al., 2018). The role of mTORC2 in thymocyte development has been studied using Rictor-deficient mice (Lee et al., 2012; Tang et al., 2012; Chou et al., 2014), but the molecular mechanism and metabolic pathways underlying mTORC2 signaling remain uncertain.

**Figure 1:**
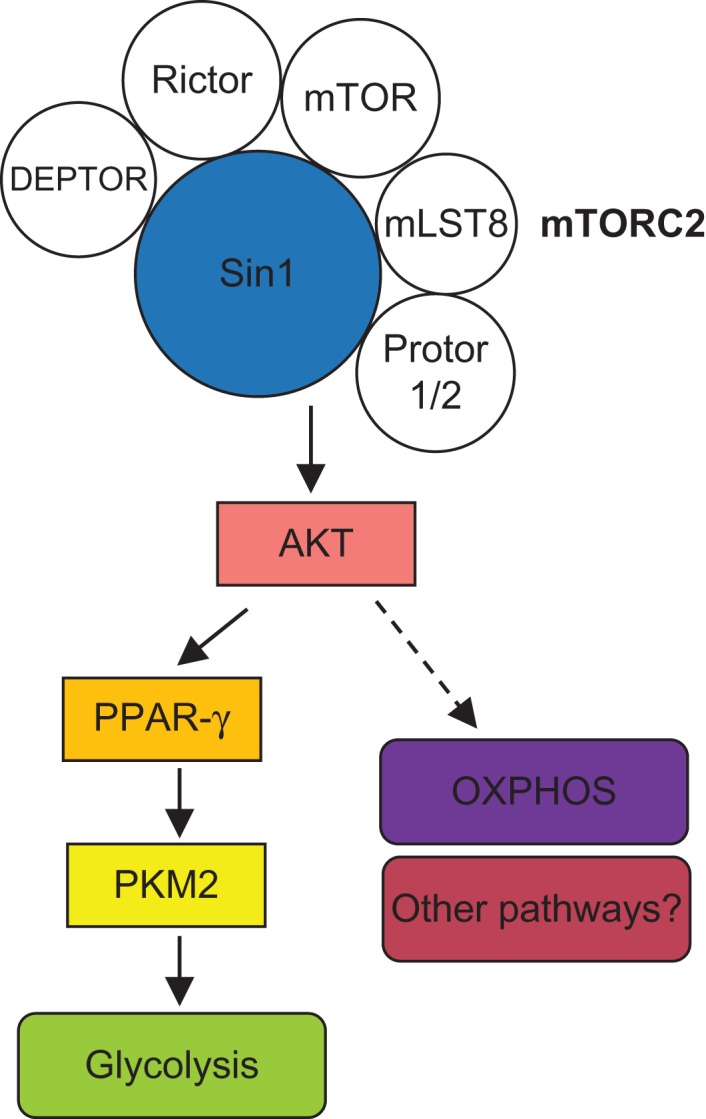
Schematic of the signaling pathways underlying Sin1–mTORC2-dependent metabolic reprogramming of early thymocyte development.

Ouyang et al. (2019) reveal a crucial role of mTORC2 in the regulation of glycolytic metabolism in early thymocyte development. The authors found that Sin1, an important component of mTORC2, promotes the proliferation of DN thymocytes in an mTORC2-dependant manner. Cre-mediated deletion of Sin1 via inducible ER-Cre or Lck-Cre system in mice results in altered development of DN subpopulations and a profound loss of DP and single-positive (SP) thymocytes. Using elegant bone marrow chimera systems, the authors reveal a crucial cell-intrinsic role of Sin1 in supporting DN thymocyte development and proliferation. Mechanistically, Sin1 augments both glycolysis and OXPHOS activities of DN thymocytes, with a particularly striking effect on the expression level of PKM2, a key rate-limiting enzyme of glycolysis by catalyzing the transfer of phosphoenolpyruvate (PEP) to pyruvate. Importantly, *in vitro* and *in vivo* treatments with a PKM2 activator partially rescue the developmental defects of Sin1-deficient thymocytes, indicating a crucial role of PKM2 in mediating Sin1–mTORC2 signaling in thymocyte development (Figure 1). Hence, Ouyang et al. (2019) establish a key mechanism by which Sin1–mTORC2 regulates DN thymocyte metabolism and development.

How does Sin1–mTORC2 regulate PKM2 expression? Previous studies in non-immune cells show that insulin or IGF treatment promotes PKM2 expression by signaling through AKT, although the specific signaling mechanisms remain poorly understood (Asai et al., 2003; Salani et al., 2015). Ouyang et al. (2019) found that the Sin1–mTORC2–AKT signaling axis promotes PKM2 expression and glycolytic metabolism in DN thymocytes by facilitating PPAR-γ nuclear translocation. Modulation of AKT or PPAR-γ activity impacts the expression of PKM2, thereby unraveling a novel Sin1–AKT–PPAR-γ–PKM2 pathway in the metabolic regulation of early thymocyte development. In addition to the defect in glycolysis, OXPHOS-related genes are also inhibited and OXPHOS activity is diminished in Sin1-deficient DN thymocytes, indicating that Sin1–mTORC2 signaling is also required for OXPHOS (Figure 1). Given the importance of OXPHOS in cell growth and metabolism, it will be interesting to explore whether and how Sin1–mTORC2-regulated OXPHOS contributes to early T cell development.

In summary, Ouyang et al. (2019) have identified Sin1 as an important regulator of the metabolism, proliferation, and development of DN thymocytes. This study identifies a previously unknown signal transduction cascade for signaling to PKM2, a key metabolic regulator essential for cell proliferation, and reveals a physiological function of Sin1–mTORC2 in the immunometabolic regulation of thymocyte development. Given the key link between thymocyte development and immune homeostasis, the conclusions from the current study may provide new insight into the therapeutic treatment of T cell lymphopenia or T cell leukemia via modulating the metabolic activity.
